# Identification through MALDI-TOF mass spectrometry and antimicrobial susceptibility profiling of bacterial pathogens isolated from sow urinary tract infection

**DOI:** 10.1080/01652176.2017.1397302

**Published:** 2017-12-07

**Authors:** Luisa Z. Moreno, Carlos E.C. Matajira, Andre P. Poor, Renan E. Mesquita, Vasco T.M. Gomes, Ana Paula S. Silva, Cristina R. Amigo, Ana Paula G. Christ, Mikaela R.F. Barbosa, Maria Inês Z. Sato, Andrea M. Moreno

**Affiliations:** a Departamento De Medicina Veterinária Preventiva e Saúde Animal, Faculdade de Medicina Veterinária e Zootecnia, Universidade de São Paulo, São Paulo, Brazil; b Companhia Ambiental do Estado de São Paulo (CETESB), São Paulo, Brazil

**Keywords:** Porcine, swine, urine, infection, MALDI-TOF, antimicrobial resistance

## Abstract

**Background:** Urinary tract infection (UTI) is a common disease in sows due to intensification of pig production. Despite direct economic losses, UTI prevalence and respective microbial identification are still poorly studied.

**Objective:** The aims of this study were to identify the causative agents of UTI in sows through MALDI-TOF MS and to characterize their antimicrobial resistance profiles.

**Materials and Methods:** Urine samples from 300 sows of three herds from São Paulo State (Brazil) were screened for UTI; suggestive samples were submitted to bacterial isolation. Species identification was performed by MALDI-TOF MS and susceptibility profiles were determined using disc diffusion method.

**Results:** 128 samples suggestive of UTI were analyzed; 48% of the animals presented UTI caused by a single pathogen, while the remaining 52% presented mixed infection. *Escherichia coli* stood out with the highest frequency among both single and mixed infections. The Gram-positive were exclusively associated with 27% of single infections. The mixed infections were further classified into 49 profiles. The high frequency of multiresistant profiles stood out for most of the studied isolates.

**Conclusions:** MALDI-TOF MS enabled the identification of rare pathogens related to UTI which may represent higher risk for porcine health, especially considering high frequency of multiresistant profiles.

## 1. Introduction

The intensification of pig production over the last few decades has led to a high degree of system productivity. However, it has also increased the infection pressure and, consequently, the manifestation of different diseases. Among the most common diseases, urinary tract infection demands attention. The pathogenic colonization of the urinary tract by one or more microorganisms, which can affect both lower and upper urinary tract, can further evolve to invasion with bacteremia and even sepsis (Merlini and Merlini 2011).

Considering that females are more predisposed to urinary tract infection (UTI), the economic losses from UTI in pig herds are significantly associated with sow health: UTI associated with postparturient urogenital disease decreases the farrowing rate and increases abortion and sow mortality (Biksi et al. 2002; Drolet and Dee 2006). Nevertheless, data on UTI prevalence and respective microbial identification are still limited.

In Brazil, the UTI incidence in commercial herds has been reported varying from 30% to 45% (Sobestiansky et al. 1995; Alberton et al. 2000; Pôrto et al. 2004). Some of the main listed causative agents are *Escherichia coli*, *Actinobacullum suis*, *Streptococcus* sp., *Staphylococcus* sp. and *Klebsiella* sp. (Pôrto et al. 2004; Brito et al. 2004; Menin et al. 2008; Merlini and Merlini 2011).

Thus, the wide variety of pathogens associated with infection and the economic losses inherent to it reinforces the need to implement a specific, rapid and low-cost method for microbial identification. Furthermore, the identification of antimicrobial resistance profiles is of great importance for treatment choice and resistance monitoring in swine herds.

Therefore, the aim of this study was to identify the causative agents of urinary tract infection in sows through MALDI-TOF MS (Matrix Assisted Laser Desorption Ionization – Time of flight mass spectrometry) and to characterize their respective antimicrobial resistance profiles.

## 2. Material and methods

### 2.1. Sample collection and UTI screening

Three hundred urine samples from sows of three full production cycle swine herds were analyzed. The herds, selected by their history of recurrent urinary infection, were located in different cities from Sao Paulo State (Brazil) and were populated by the same genetic lineage (Landrace, Large White and Pietrain crossbred). Sows’ midstream urine samples were taken in using a sterile universal sample collector after spontaneous micturition in the first hour of morning. The urine samples with characteristics suggestive of urinary tract infection based on dipstick test screening results (leukocyturia, nitrite presence, proteinuria and pH > 7.5) were selected for further analysis.

### 2.2. Bacterial isolation

The urine samples (10 mL) were centrifuged at 4,000 × *g* for 10 min and the obtained pellet was plated in MacConkey, Chromagar Orientation™ and blood agar (5% defibrinated sheep blood) (Difco-BBL, Sparks, MD, USA). The agar plates were incubated under aerobic and microaerophilic conditions for 24–48 h at 37 °C. Each colony of interest was maintained at −86 °C in brain-heart infusion (BHI) medium (Difco, Sparks, MD, USA) with 30% of glycerol, supplemented with fetal calf serum (5%) when necessary for fastidious pathogens, for further analysis.

### 2.3. Bacterial identification

The selected colonies were initially screened by matrix-assisted laser desorption ionization–time of flight mass spectrometry (MALDI-TOF MS) identification. MALDI-TOF MS sample preparation, data processing and analysis were done as previously described by Hijazin et al. (2012). Mass spectra were acquired using a Microflex™ mass spectrometer (Bruker Daltonik) and identified with manufacturer's software MALDI BioTyper™ 3.0. Standard Bruker interpretative criteria were applied; scores ≥ 2.0 were accepted for species assignment and scores ≥ 1.7 but ≤ 2.0 for genus identification.

For the species confirmation, specifically for the strains from *Streptococcus*, *Aerococcus*, *Globicatella* and *Corynebacterium* genus, 16S rRNA gene sequencing was performed using Twomey et al. (2012) primers. The obtained sequences were compared to the GenBank nucleotide non-redundant database through BLAST analysis.

### 2.4. Antimicrobial susceptibility profiling

Susceptibility profiles were determined using disc diffusion method according to the standardized VET01-A4 supplement (CLSI 2013). The antimicrobial agents tested included: ampicillin (10 µg), ceftiofur (30 µg), sulfisoxazole (300 µg), trimethoprim-sulfamethoxazole (1.25/23.75 µg), tetracycline (30 µg), enrofloxacin (5 µg), florfenicol (30 µg), spectinomycin (100 µg), and gentamycin (10 µg); for Gram-negative pathogens were also included amoxicillin/clavulanic acid (20/10 µg), nalidixic acid (30 µg), norfloxacin (10 µg), ciprofloxacin (5 µg) and streptomycin (10 µg), while the Gram-positive bacteria were also tested for penicillin (10 U), doxycycline (30 µg), neomycin (30 µg), clindamycin (2 µg) and tilmicosin (15 µg).

As quality control, the *Escherichia coli* ATCC 25922 and *Staphylococcus aureus* ATCC 25923 reference strains were used. The interpretative breakpoints were obtained in the supplements VET01S (CLSI 2015), VET06 (CLSI 2017) and M100-S19 (CLSI 2009).

### 2.5. Statistical analysis

The mixed infections were classified into profiles considering the identified species. The resistance profiles were determined according to the observed results (susceptible, intermediary, resistant) for the studied antimicrobials. The cluster analysis for both mixed infection and resistance profiles was performed with Bionumerics 7.6 (Applied Maths NV, Sint-Martens-Latem, Belgium); profiles were analyzed as categorical data using *different values* coefficient and Ward method. The multiresistance was determined according to Schwarz et al. (2010).

## 3. Results

A total of 128 urine samples with characteristics suggestive of urinary tract infection were analyzed. Among these, 31 samples originated from herd 1 (H1), 72 from herd 2 (H2) and 25 from herd 3 (H3). 48% (62/128) of the animals presented urinary infection caused by a single pathogen, while the remaining 52% (66/128) presented mixed infection. Two to four different bacterial species were isolated from samples of animals presenting mixed infection (Table 1).

**Table 1.** Infection characterization of studied urine samples with characteristics suggestive of urinary tract infection.

		Herd		
Bacterial infection	*N* (%)	H1	H2	H3
One species	62 (48,4)	17 (54.8)	28 (38.9)	17 (68.0)
Two species	39 (30.5)	9 (29.1)	24 (33.3)	6 (24.0)
Three species	25 (19.5)	5 (16.1)	18 (25.0)	2 (8.0)
Four species	2 (1.6)	0	2 (2.8)	0
Total	128 (100)	31 (100)	72 (100)	25 (100)

Seven Gram-negative and 25 Gram-positive species associated with urinary infection were identified in this study (Table S1). Among the bacterial species isolated from single infection (Table 2), *Escherichia coli* stood out with the highest frequency (71% - 44/62) and Gram-positive agents were isolated in 27% (17/62) of single infections, in which *Streptococcus hyovaginalis* predominated (35%).

**Table 2.** Bacterial species associated with single urinary infection.

		Herd		
Species	*N* (%)	H1	H2	H3
*Escherichia coli*	44 (71.0)	6 (35.3)	23 (82.1)	15 (88.2)
*Streptococcus hyovaginalis*	6 (9.7)	6 (35.3)	–	–
*Enterococcus faecalis*	3 (4.8)	2 (11.8)	–	1 (5.9)
*Enterococcus faecium*	2 (3.2)	–	1 (3.6)	1 (5.9)
*Globicatella sulfidifaciens*	2 (3.2)	1 (5.9)	1 (3.6)	–
*Aerococcus viridans*	1 (1.6)	–	1 (3.6)	–
*Corynebacterium confusum*	1 (1.6)	1 (5.9)	–	–
*Providencia rettgeri*	1 (1.6)	–	1 (3.6)	–
*Streptococcus dysgalactiae*	1 (1.6)	–	1 (3.6)	–
*Streptococcus pluranimalium*	1 (1.6)	1 (5.9)	–	–
Total	62 (100)	17 (100)	28 (100)	17 (100)

**Figure 1.**
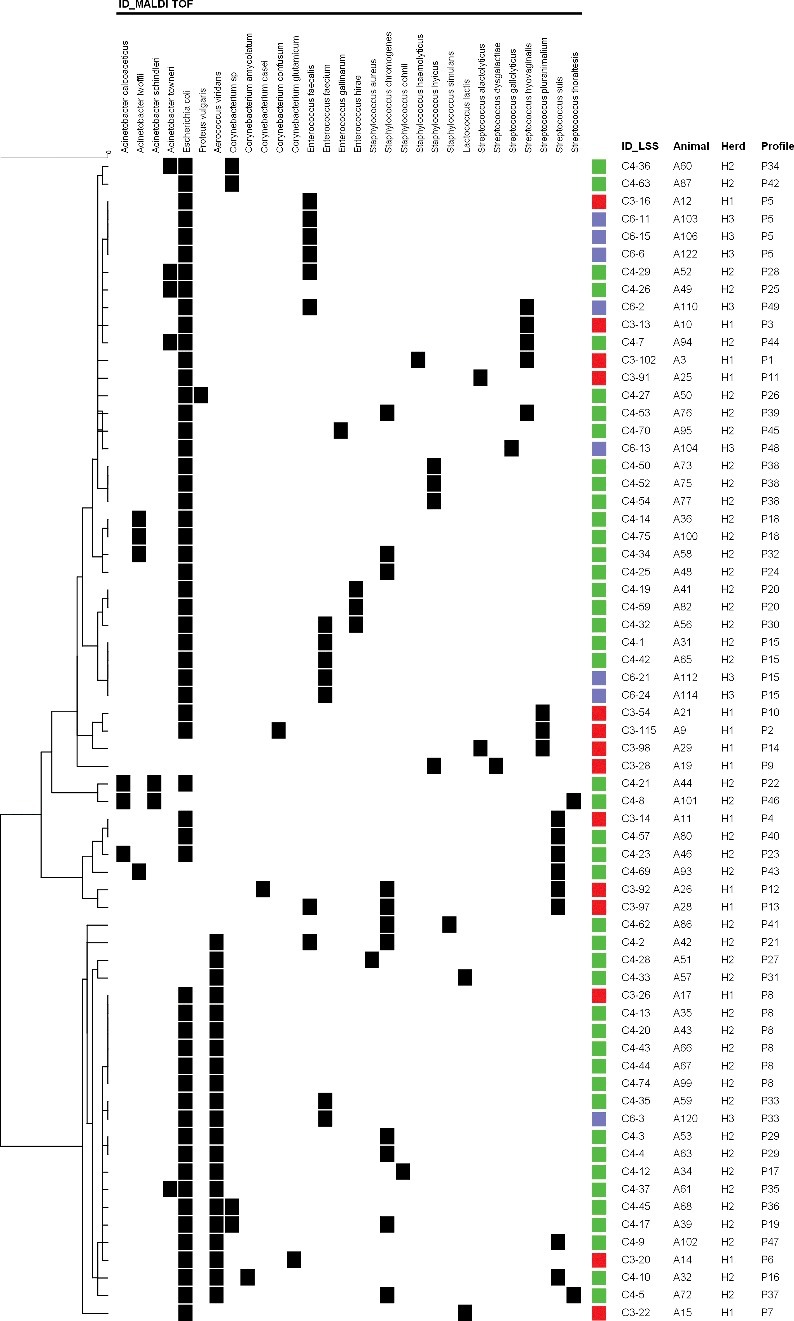
Mixed infections cluster analysis with identification and characterization of infection profiles. The colors indicate the herd of origin (green – H2, red – H1, blue – H3).

Considering the animals with mixed infections, *E. coli* was present in 85% (56/66), of which 93% (52/56) were associated to a Gram-positive bacterial species. Only 12% (8/66) of mixed infections were caused exclusively by Gram-positive bacteria. The mixed infections were further classified into 49 profiles (P1 – P49) (Figure 1). The higher frequency profiles were composed of *A. viridans* and *E. coli *(P8) (9%), *E. coli* and *E. faecalis* (P5) (6%), and *E. coli* and *E. faecium* (P15), present in at least two of the studied herds (6%) (Figure 1).

**Figure 2.**
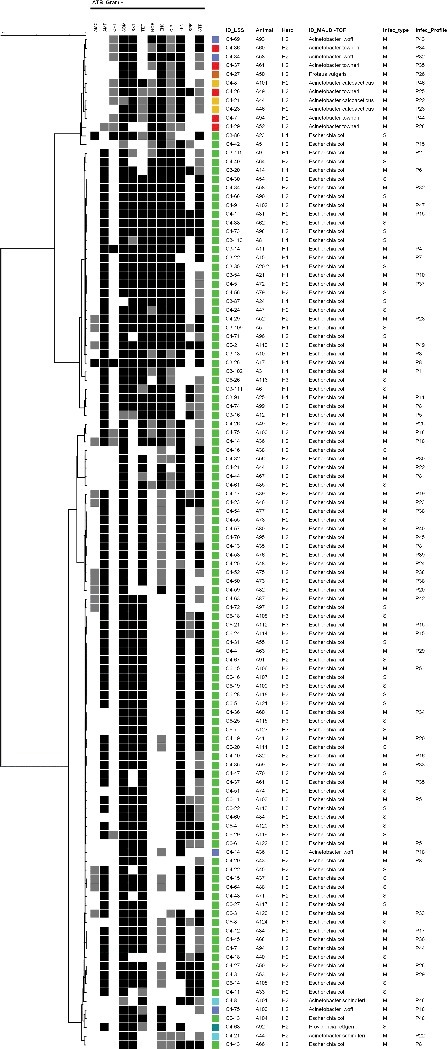
Resistance profiles cluster analysis of studied Gram-negative pathogens. The grey scale (black, grey and white) corresponds to resistant, intermediate and sensitive status, respectively. The colored squares indicate the different Gram-negative bacterial species.

In regard to the antimicrobial susceptibility of Gram-negative pathogens, high resistance rates were observed for tetracycline (78.4%), florfenicol (84.5%), sulfonamides (95.7% and 70.7%) and streptomycin (50.9%) (Table 3). Among the 116 Gram-negative isolates studied, multiresistance was detected in 95.7% (Table 4). Most *E. coli* isolates were classified as multiresistant (98%) and even though *Acinetobacter* spp. isolates were also multiresistant, they presented susceptibility to β-lactams and tetracycline (Table 4, Figure 2).

**Table 3.** *In vitro* resistance rates of Gram-negative pathogens (N = 116) – N (%).

Antimicrobial	Susceptible	Intermediary	Resistant
Amoxicillin/clavulanic acid	99 (85.3)	15 (12.9)	2 (1.7)
Ampicillin	31 (26.7)	1 (0.9)	84 (72.4)
Ceftiofur	102 (87.9)	11 (9.5)	3 (2.6)
Sulfadimethoxine	1 (0.9)	4 (3.4)	111 (95.7)
Trimethoprim/ sulpham^a^.	31 (26.7)	3 (2.6)	82 (70.7)
Tetracycline	15 (12.9)	10 (8.6)	91 (78.4)
Norfloxacin	70 (60.3)	12 (10.3)	34 (29.3)
Enrofloxacin	35 (30.2)	27 (23.3)	54 (46.6)
Ciprofloxacin	63 (54.3)	24 (20.7)	29 (25.0)
Florfenicol	15 (12.9)	3 (2.6)	98 (84.5)
Spectinomycin	85 (73.3)	11 (9.5)	20 (17.2)
Streptomycin	12 (10.3)	45 (38.8)	59 (50.9)

a Trimethoprim/sulphamethoxazole.

**Table 4.** Gram-negative pathogens resistance profile distribution according to the number of resistant antimicrobial classes.

	Resistance profile			
Species	1 - 2 classes	3 - 4 classes	≥ 5 classes	Total
*Acinetobacter calcoaceticus*	-	3 (100)	-	3 (100)
*Acinetobacter lwoffii*	1 (25.0)	3 (75.0)	-	4 (100)
*Acinetobacter schindleri*	1 (50.0)	1 (50.0)	-	2 (100)
*Acinetobacter towneri*	1 (20.0)	4 (80.0)	-	5 (100)
*Escherichia coli*	2 (2.0)	42 (42.0)	56 (56.0)	100 (100)
*Proteus vulgaris*	-	1 (100)	-	1 (100)
*Providencia rettgeri*	-	1 (100)	-	1 (100)
Total	5 (4.3)	55 (47.4)	56 (48.3)	116 (100)

The resistance profiles cluster analysis enabled the differentiation of three main groups in which the first is composed of most of *Acinetobacter* species and *P. vulgaris*, while the second consists mainly of *E. coli* isolates (Figure 2). No relation between resistance profiles and infection (single or mixed infection) was observed among the studied Gram-negative pathogens.

Regarding the susceptibility of Gram-positive pathogens, they presented more homogeneous resistance rates (Table 5) with highest resistance observed for aminoglycosides and tylosin. Among the 108 studied isolates, 79% were characterized as multiresistant (Table 6) with highlight for *Aerococcus viridans* and the *Staphylococcus* and *Streptococcus* species. Interestingly, the *Enterococcus* genus was the least resistant among the studied Gram-positive pathogens, with seven *E. faecalis* isolates completely sensitive for all tested antimicrobials.

**Table 5.** *In vitro* resistance rates of Gram-positive pathogens (*N* = 108) – *N* (%).

Antimicrobial	Susceptible	Intermediary	Resistant
Penicillin	59 (54.6)	6 (5.6)	43 (39.8)
Ampicillin	60 (55.5)	3 (2.8)	45 (41.7)
Ceftiofur	60 (55.5)	6 (5.6)	42 (38.9)
Tetracycline	52 (48.1)	6 (5.6)	50 (46.3)
Enrofloxacin	42 (38.9)	16 (14.8)	50 (46.3)
Sulfadimethoxine	79 (73.1)	3 (2.8)	26 (24.1)
Trimet/sulfamet^a^	65 (60.2)	1 (0.9)	42 (38.9)
Florfenicol	50 (46.3)	12 (11.1)	46 (42.6)
Clindamycin	57 (52.8)	13 (12.0)	38 (35.2)
Gentamycin	46 (42.6)	3 (2.8)	59 (54.6)
Neomycin	55 (50.9)	3 (2.8)	50 (46.3)
Spectinomycin	55 (50.9)	18 (16.7)	35 (32.4)
Tylosin	39 (36.1)	–	69 (63.9)

a Trimethoprim/sulfamethoxazole.

**Table 6.** Gram-positive pathogens resistance profile distribution according to the number of resistant antimicrobial classes.

	Resistance profile			
Species	1–2 classes	3–4 classes	≥5 classes	Total
*Aerococcus viridans*	–	7 (31.8)	15 (68.2)	22 (100)
*Corynebacterium amycolatum*	–	1 (100)	–	1 (100)
*Corynebacterium casei*	–	1 (100)	–	1 (100)
*Corynebacterium confusum*	–	1 (50.0)	1 (50.0)	2 (100)
*Corynebacterium glutamicum*	–	1 (100)	–	1 (100)
*Corynebacterium sp*	1 (20.0)	4 (80.0)	–	5 (100)
*Enterococcus faecalis*	4 (36.4)	–	–	11 (100)
*Enterococcus faecium*	6 (66.7)	3 (33.3)	–	9 (100)
*Enterococcus gallinarum*	–	1 (100)	–	1 (100)
*Enterococcus hirae*	3 (100)	–	–	3 (100)
*Globicatella sulfidifaciens*	1 (50.0)	1 (50.0)	–	2 (100)
*Lactococcus lactis*	1 (50.0)	–	1 (50.0)	2 (100)
*Staphylococcus aureus*	–	1 (100)	–	1 (100)
*Staphylococcus chromogenes*	–	7 (63.6)	4 (36.4)	11 (100)
*Staphylococcus cohnii*	–	1 (100)	–	1 (100)
*Staphylococcus haemolyticus*	–	–	1 (100)	1 (100)
*Staphylococcus hyicus*	–	3 (75.0)	1 (25.0)	4 (100)
*Staphylococcus simulans*	–	–	1 (100)	1 (100)
*Streptococcus alactolyticus*	–	–	2 (100)	2 (100)
*Streptococcus dysgalactiae*	–	–	2 (100)	2 (100)
*Streptococcus gallolyticus*	–	–	1 (100)	1 (100)
*Streptococcus hyovaginalis*	–	–	10 (100)	10 (100)
*Streptococcus pluranimalium*	–	–	4 (100)	4 (100)
*Streptococcus suis*	–	3 (37.5)	5 (62.5)	8 (100)
*Streptococcus thoraltensis*	–	–	2 (100)	2 (100)
Total	16 (14.8)	35 (32.4)	50 (46.3)	108 (100)

**Figure 3.**
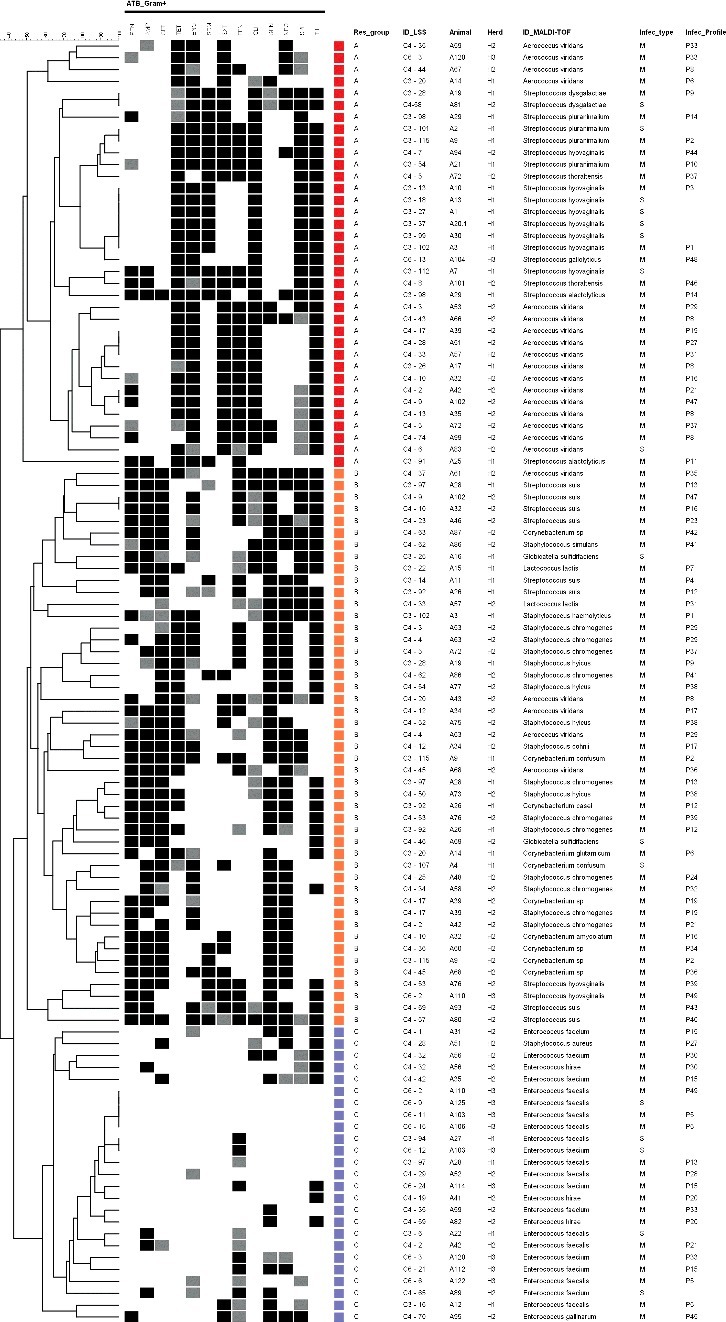
Resistance profiles cluster analysis of studied Gram-positive pathogens. The grey scale (black, grey and white) corresponds to resistant, intermediate and sensitive status, respectively. The colored squares indicate the detected resistance groups (red – A, orange – B, blue – C).

The resistance profiles cluster analysis enabled the differentiation of three groups (A–C) (Figure 3), wherein the A and B groups are composed of most of the multiresistant isolates. Group A comprises 36 isolates, all of which are resistant to more than four antimicrobial classes, including most of *A. viridans* and *Streptococcus* species, with exception of *S. suis* that were separated in group B that consists of 46 isolates, with 96% multiresistant. These include the *S. suis*, *Corynebacterium* and 95% of *Staphylococcus* isolates. While group A is characterized by resistance to tetracycline, enrofloxacin, clindamycin and tylosin, with variable resistance to sulfonamides and florfenicol according to the identified genus and species, group B is mainly characterized by β-lactams, aminoglycosides and tylosin resistance. The remaining Gram-positive isolates comprise group C that includes the few susceptible *E. faecalis* and the less-resistant strains.

## 4. Discussion

The predominance of *E. coli* among studied isolates, in both single and mixed infections, corroborates previous studies that also detected *E. coli* as the most frequent bacteria in sows’ UTI cases (Brito et al. 2004; Menin et al. 2008; Merlini and Merlini 2011; Mazutti et al. 2013). The high frequency of *E. coli* in mixed infections should not be disregarded or merely considered as contamination, since over 80% of the studied *E. coli* isolates were characterized as multiresistant and 93% of them presented at least one virulence gene related to urinary tract infections, among *foc*H*, pap*C*, sfa, afa, hly*A*, iuc*D and *cnf*1 genes (data not shown).

The high frequency of *Streptococcus* sp. and *Streptococcus*-like bacteria, such as *A. viridans*, also corroborates previous findings considering that most studies only reported the identification of *Streptococcus* sp. with hemolysis differentiation since they relied solely on traditional isolation and biochemistry methods for bacterial identification (Menin et al. 2008; Merlini and Merlini 2011; Mazutti et al. 2013).

The MALDI-TOF MS technique has already been endorsed for the identification of several microorganisms (Biswas and Rolain 2013; Singhal et al. 2015), including rare bacterial species implicated in human and animal infectious disease (Seng et al. 2013). Considering the variety of Gram-positive species identified in this study, especially of *Streptococcus*, *Staphylococcus* and *Enterococcus*, the MALDI-TOF MS represents not only a high throughput solution but also a reliable alternative to biochemical tests, which are not only laborious but often provide dubious results.

Since most studies of antimicrobial susceptibility characterization mainly focus on *E. coli* or rarely on *Streptococcus* sp., resistance data regarding most of the Gram-positive bacteria identified in this study is scarce. For *E. coli*, our results agree with the reported high multiresistance frequency, with elevated levels of tetracycline, florfenicol and sulfonamides; however, the observed streptomycin high resistance rate differ from previous studies (Costa et al. 2008; Menin et al. 2008; Hancock et al. 2009).

In regard to *Streptococcus* sp., Menin et al. (2008) described high resistance to aminoglycosides and fluoroquinolone with greater susceptibility to β-lactams. In this study, we observed variability of resistance profiles according to the identified *Streptococcus* species; nevertheless, all isolates were characterized as multiresistant. The multiresistant profiles stand out for most Gram-positive bacteria while the *Enterococcus* genus was highlighted as the most susceptible among the studied isolates.

Therefore, with the improvement of microbiological methods for proper diagnosis and bacterial identification, underestimated pathogens are related to urinary infection, which is still poorly studied in farm animals. These pathogens may represent a potential risk for porcine health and should be properly identified by veterinary diagnostic laboratories. Furthermore, characterization of antimicrobial resistance profiles is of significant importance not only for animal treatment but also for resistance monitoring which could be applied to both human and animal health promotion programs.
